# The New Treatment for Tuberculosis

**Published:** 1913-07

**Authors:** Stanley Sevier Warren


### EDITORIAL DEPARTMENT.

**DR. F. E. DANIEL, Editor** **DR. R. H. L. BIBB, Associate Editor**

DEPARTMENTS { EUGENICS: Drs. M. Duggan and T. Y. Hull.

WOMAN'S DEPARTMENT: Mrs. F. E. Daniel.

1885-1913, Volume 1 to Volume 29, under one—the original—management. This issue begins the twenty-ninth year in the service of the profession. That it has survived the death and burial of numerous "journals" in that time testifies to the generous support bestowed upon it both by the profession and the advertisers who cater to them. I take off my hat twenty-nine times and bow twenty-nine times, and here she goes, off for the twenty-ninth round.

---

THE NEW TREATMENT FOR TUBERCULOSIS.—In our June issue we reproduced a most remarkable paper by Dr. S. S. Warren of San Angelo, in which he gives his experience with carbolized vaseline in the treatment of tuberculosis, and his ideas of its rationale. The proof of the pudding, you know, is in the eating. It matters not how it does it, the treatment in Dr. Warren's hands has been remarkably successful. You know we never learned to fly until we abandoned the balloon idea, a fallacy, and struck out on a new line, the opposite. May it not be that we are on the wrong track in seeking a cure for tuberculosis via the serum route? At any rate, Dr. Warren has nothing to sell and freely gives his treatment, which I append. It certainly is worthy of trial. It can do no harm and costs little. Should this prove to be a real discovery (and it looks like it) Dr. Warren will go down in history as a benefactor of his race and his discovery should receive recognition at the hands of the government, as well as the profession.

Here are the directions for use:

*Liquid Carbolated Petroleum** (*Phenol Petrolatum*).—Liquid Carbolated Petrolatum is, as is name indicates, carbolic acid incorporated in liquid petrolatum.

It is prepared in ½% and 1% solution.

The treatment is as follows:

Fifteen minims of the ½% is given daily for 15 days; and 15 minims of the 1% every second day for 30 days.

It is given subcutaneously, in the back, with an ordinary hypodermic needle.

---

*Phenol-petrolatum can be purchased from Parke-Davis of Kansas City, Mo., in ½% and 1% solution in 8 oz. bottles.

There should be an interval of from 10 to 20 days between first and second course.

Where the expectoration diminishes rapidly the treatment should only be given every second day.

The temperature changes are characteristic in that the highest temperature of the day will gradually work itself down until the noon-day finds it at its highest.

The febrile symptoms eventually disappear, bring the 7 a. m. or subnormal temperature to normal.

When this condition is established, I feel justified in saying that the pathological processes are completely arrested, and the natural physiological functions will soon enable nature to complete the cure.

I strongly advise that you prohibit your patients from taking raw eggs, as I find in all egg eating patients a gastro-intestinal complication that in every case accounts for the high fevers that accompany advanced tuberculosis.

The only contraindication for its use is nephritis.

As a preliminary treatment I would suggest small doses of calomel and phenolphthalein.

STANLEY SEVIER WARREN, M. D.

